# Practical Method for Freezing Buck Semen

**DOI:** 10.3390/ani12030352

**Published:** 2022-02-01

**Authors:** Jane M. Morrell, Pongpreecha Malaluang, Theodoros Ntallaris, Anders Johannisson

**Affiliations:** Clinical Sciences, Swedish University of Agricultural Sciences (SLU), P.O. Box 7054, SE-75007 Uppsala, Sweden; pongpreecha.malaluang@slu.se (P.M.); theodoros.ntallaris@slu.se (T.N.); anders.johannisson@slu.se (A.J.)

**Keywords:** goat sperm cryopreservation, removal of seminal plasma, soy lecithin semen extender, plasma membrane integrity, chromatin integrity

## Abstract

**Simple Summary:**

Goat semen was previously considered to be problematic to freeze because of reactions between the semen and the components of the freezing media that were available at the time. However, there have been reports of several successful attempts to freeze goat semen in recent decades using various protocols, resulting in usable post-thaw sperm samples. In the present study, we adapted some of these methods to suit the particular conditions under which we had to work. We were able to produce thawed samples with acceptable sperm quality which were sent to a sperm bank for long-term storage.

**Abstract:**

Although several protocols for cryopreserving buck semen are described in the literature, they differ widely in factors such as season and method of semen collection, extender and sperm concentration. Therefore, choosing a protocol that is suitable for a particular on-farm situation can be problematic. In the present study, semen was collected by artificial vagina from seven bucks on a farm located approximately 90 minutes’ drive away from the laboratory, about 6 weeks before the start of the goat breeding season. The semen was immediately extended in warm semen extender containing soy lecithin and was placed in an insulated box with a cold pack for up to 4 h, during semen collection from the remaining bucks and subsequent transport to the laboratory. Following centrifugation at 4 °C and resuspension in the soy lecithin extender to a sperm concentration of 800 × 10^6^ spermatozoa/mL, 0.25 mL plastic straws were filled and frozen in racks 4 cm above the surface of liquid nitrogen. This simple protocol resulted in an acceptable post-thaw quality for all seven bucks, with a mean post-thaw motility of 55 ± 21% and mean fragmented chromatin of 3.27 ± 1.39%. Normal sperm morphology was >90% in all ejaculates. The semen was sent to a gamete bank for long-term storage.

## 1. Introduction

There are relatively few papers on freezing goat semen compared to the considerable volume of literature available for species such as the bull, but the reports that are available for buck semen present a bewildering array of protocols. Some examples are given in Table 1. These protocols differ in timing of semen collection (in or out of the breeding season), semen collection method (artificial vagina or electroejaculation), type of semen extender (containing egg yolk or not), whether or not the seminal plasma should be removed prior to freezing, how long the semen should be equilibrated prior to freezing (2 h, 4 h), what the final sperm concentration should be (ranging from 50 × 10^6^/mL to 1000 × 10^6^/mL), pellet or straw, size of straw (0.25 mL or 0.5 mL), and the method of freezing (dry ice for pellets, vapor freezing or controlled rate freezing for straws). All of these factors could influence post-thaw sperm quality. The range in sperm concentration among the various studies may reflect the intended use of the semen: artificial insemination (AI), in vitro fertilization (IVF), laparoscopic insemination, etc., but could have an effect on the ability of the spermatozoa to survive freezing. These different studies reflect the wide variety of conditions under which buck semen might conceivably be collected for freezing, but each of the factors involved could have an effect on sperm cryo-survival. Therefore, it is not easy to identify a practical on-farm method for freezing buck semen for use in AI.

The cryo-extenders that were used originally for freezing mammalian semen contained skimmed milk or egg yolk or both. These substances present a problem for processing buck semen since buck seminal plasma contains a protein that coagulates egg yolk [9]. Similarly, SUBIII, a protein from the goat bulbourethral gland, hydrolyses residual triglycerides in the medium, producing fatty acids that are toxic to spermatozoa [10]. However, extenders are now available that are not based on egg yolk or skimmed milk, such as Andromed which contains soy lecithin. Some studies suggest that removal of the seminal plasma by washing is not necessary if the semen is to be frozen in a soy lecithin-containing medium such as Andromed [4]; other protocols remove the seminal plasma before freezing the semen in Andromed [1].

In the present study, the objective was to collect and freeze buck semen for gamete banking purposes. The intention was that the sperm samples could be used in the future for artificial insemination (AI) of goats in Sweden, preferably by the vaginal deposition method—the so-called “shot-in-the-dark” technique [1]. The goats were on a farm at some distance from the semen laboratory, necessitating a 90 min drive; they were available for a short period of time prior to the start of the breeding season and were to be returned to their farm of origin after sufficient ejaculates had been collected for sperm banking. The protocol used was developed from other published protocols.

## 2. Materials and Methods

### 2.1. Animals

Seven Landrace bucks were transported to a goat farm in Sweden that was certified to be free of caprine arthritis/encephalitis virus (CAEV), where they were held in isolation for 6 weeks prior to semen collection according to the regulations laid down by the Swedish Board of Agriculture. They were fed on commercial pellets and good quality hay and were bedded on straw. While in isolation, serum samples were taken from the bucks for testing for antibodies to Border Disease virus and also for CAEV. The animals were housed and kept according to national and international regulations for the husbandry of livestock species.

### 2.2. Semen Handling

Semen collection took place at the beginning of September using an artificial vagina after introducing the bucks individually to an estrous doe. Two ejaculates were collected from each buck, with a period of rest (approximately 1 h) between the two collections. Immediately after collection, the ejaculates were extended with warm (30 °C) extender (Andromed; Minitüb International, Tiefenbach, Germany) up to the 10 mL graduation mark in a 50 mL centrifuge tube and were immediately transferred to a large insulated box containing cold packs. The semen tubes were placed on a layer of paper towel to avoid direct contact with the cold packs. Once all the ejaculates had been collected, the semen was transferred to the laboratory at the Swedish University of Agricultural Sciences, Uppsala, Sweden, for further processing. The time for 14 collections was approximately 2 h; the journey to the laboratory was a further 90 min.

Aliquots (5 µL) of semen were assessed for sperm motility by Computer Assisted Sperm Analysis (SpermVision, Minitüb International). An aliquot (5 µL) was placed on a glass slide on the warm stage (38 °C) of an Olympus BX 51 microscope. The kinematics were measured in 8 fields, containing at least 1000 spermatozoa, using the settings for ram spermatozoa determined in our laboratory, at a frame rate of 60/s. Particles with an area ranging from 20 to 100 μm^2^ were included in the analysis. The following kinematics were measured: total motility (TM, %), progressive motility (PM, %), average path velocity (VAP; µm/s), curvilinear velocity (VCL; µm/s straight line velocity (VSL; µm/s), the ratios STR (VSL/VAP), LIN (VSL/VCL) and WOB (VAP/VCL), amplitude of lateral head displacement (ALH; µm), and beat cross frequency (BCF; hz). Spermatozoa were identified as immotile (BCF < 0.2; VSL < 0.2), hyper-motile (VCL > 80; LIN < 0.65; ALH > 6.5), and progressively motile (STR > 0.5 and LIN > 0.35).

Sperm concentration was measured using the Nucleo-counter SP-100 (Chemometec, Allerød, Denmark). Briefly, 5 µL of semen were mixed with 0.5 mL Reagent S100 before loading into cassettes containing propidium iodide (PI) and placing in the reader. Fluorescence from the stained sperm nuclei was converted to sperm concentration.

The remaining semen was immediately centrifuged (800× *g* for 10 min) at 4 °C. The total sperm number (obtained from volume x concentration) was used to calculate the final volume of extender that would be needed to give a sperm concentration of 800 × 10^6^/mL. After removing the supernatant, the sperm pellets were resuspended in the calculated volume of extender at 4 °C. Straws were filled with 200 µL of extended semen and the ends were heat-sealed. Thus, each straw contained an insemination dose of 200 × 10^6^ spermatozoa, with an air bubble included to prevent the cotton plug at one end from being expelled during temperature changes.

The straws were placed on a rack 4 cm above the surface of liquid nitrogen for 10 min before being plunged into liquid nitrogen and transferred to a storage vessel. The total time from collection to freezing was approximately 6 h, during which time the semen was being cooled and then maintained at 4 °C. After 24 h in liquid nitrogen, one straw from each batch was thawed in a water bath (37 °C for 12 s) for sperm quality assessment.

### 2.3. Post-Thaw Semen Analysis

#### 2.3.1. Membrane Integrity

Membrane integrity was evaluated using a combination of the fluorochromes SYBR-14 and PI (Live-Dead Sperm Viability Kit L-7011; Invitrogen, Eugene, OR, USA). Sperm samples were diluted to 2 × 10^6^ spermatozoa/mL with Buffer B (patent applied for; SLU, Uppsala, Sweden); 0.6 µL of SYBR-14 (1:50 in Buffer B) and 3 µL of PI were added. The tubes were kept in the dark at 38 °C for 10 min. Samples were analysed using a FacsVerse flow cytometer (BDBiosciences; Franklin Lakes, NJ, USA) equipped with standard optics. Excitation was induced by a blue laser (488 nm). Forward scatter (FSC) and side scatter were collected. Green fluorescence (FL1) from SYBR 14 was detected with a 507 nm long-pass dichroic mirror, followed by a band-pass filter (527/32 nm), while red fluorescence (FL3) from PI was measured using a 665 nm long-pass dichroic mirror, followed by a band-pass filter (700/54 nm). A compensation of 24.12% was used for FL3-FL1, while no compensation was used for FL1-FL3. From each sample, measurements from 50,000 events were collected and quantified as percentages.

#### 2.3.2. Sperm Chromatin Structure Assay

Aliquots of sperm samples (20 µL) were mixed 1:1 with TNE buffer (Tris-sodium chloride-EDTA; 0.15 mol/L NaCl, 0.01 mol/L Tris-HCl, 1 mmol/L EDTA, pH 7.4), snap-frozen in liquid nitrogen and stored at −80 °C until required for analysis. After thawing the samples on ice, aliquots (10 μL) were extended with TNE buffer (90 μL) and subjected to partial DNA denaturation in situ with 0.2 mL detergent solution (0.17% Triton X-100, 0.15 mol/L NaCl, and 0.08 mol/L HCl; pH 1.2). The samples were stained with acridine orange (0.6 mL; 6 μg/mL in 0.1 mol/L citric acid, 0.2 mol/L Na_2_HPO_4_, 1 mmol/L EDTA, 0.15 mol/L NaCl; pH 6.0). Within 3–5 min, the samples were analysed using a flow cytometer (FACSVerse, BDBiosciences). For each sample, at least 10,000 events were analyzed at a speed of 200 cells/s after excitation with a blue laser (488 nm). Both FSC and SSC were collected. The FL1 (green fluorescence) was collected through a 507 nm long-pass dichroic mirror, followed by a band-pass filter (527/32 nm); FL3 (red fluorescence) was collected using a 665 nm long-pass dichroic mirror, followed by a band-pass filter (700/54 nm). The DNA Fragmentation Index (%DFI—the ratio of cells with denatured, single-stranded DNA to total cells acquired) was calculated for each sample using FCS Express version 5 (De Novo Software, Pasadena, CA, USA).

#### 2.3.3. Sperm Morphology

The morphology evaluation was carried out by trained personnel in the Swedish Sperm Reference Laboratory at SLU. Smears of thawed semen were stained with carbolfuchsin-eosin after air-drying [11]. Five hundred spermatozoa were evaluated using ×1000 magnification with oil immersion. Individual spermatozoa were classified as having normal morphology if no abnormality was detected. A further aliquot of semen was fixed in formol-saline; a wet mount was prepared for evaluation of 200 spermatozoa at ×1000 magnification. The proportion of normal spermatozoa in the sample was calculated from the average value of the stained smear and the wet mount.

#### 2.3.4. Statistical Analysis

All statistical analyses were performed with SAS software (version 9.4; SAS Institute Inc., Cary, NC, USA). Unless otherwise specified, data were analysed using the MIXED procedure for linear mixed models. The model included the fixed effect of ejaculate (two classes; first or second), treatment (two classes; fresh or frozen), and their interaction. The random effect of buck was included in the model. The residuals from the observations generated from the mixed models were tested for normal distribution. Least squares means (LSMeans ± sem) estimated by the models were adjusted using Scheffé adjustment for multiple post-ANOVA comparisons and compared. 

Associations between pre-freeze and post-thaw motility, and also between post-thaw motility and membrane integrity, were analysed using Pearson correlation after testing for normality. Differences with *p* ≤ 0.05 were considered significant. Differences with 0.05 < *p* ≤ 0.10 were considered as trends. 

## 3. Results

Semen volume ranged from 0.75 to 2 mL; initial motility (approximately 2–4 h after collection) was 91–95% (Table 2).

Post-thaw motility ranged from 0 (one batch) to 84%, with all but two batches having post-thaw motility of >40% (Table 3). Motility in the fresh sample was not related to post-thaw motility (r = 0.28; NS) and there was considerable variation between bucks. 

**Figure 1:**
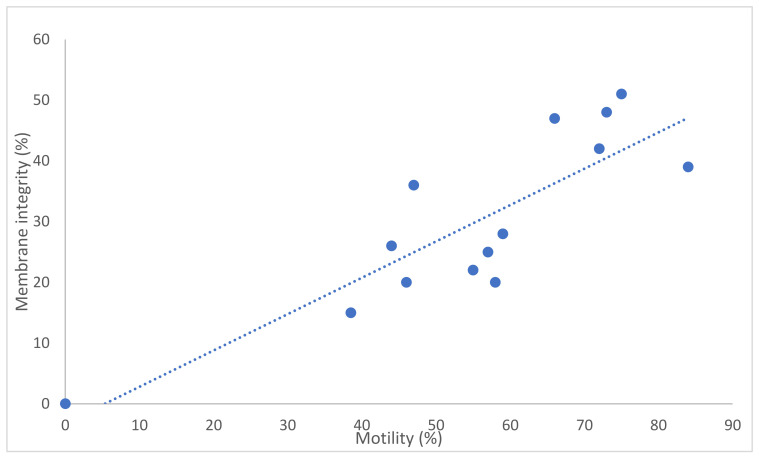
Association between sperm motility and membrane integrity for thawed buck spermatozoa (*n* = 14 ejaculates). Note: correlation coefficient r = 0.85; *p* < 0.001.

The number of straws produced per ejaculate varied from 10 to 35. Post-thaw membrane integrity varied from 15% to 48% (Table 4). There was a good correlation between total motility and membrane integrity (r = 0.85; *p* < 0.001) although the actual values were different, with motility being considerably higher (approximately 25%) than membrane integrity (Figure 1). There were no differences between the post-thaw sperm quality of the first and second ejaculates except for chromatin integrity; the DNA fragmentation index was higher in the second ejaculate than in the first.

Sperm morphology was good for all the bucks with at least 90% normal morphology in all ejaculates. The few defects that were observed were mostly tail defects, such as coiled tails. Sperm chromatin integrity was also very good, with an overall mean value of 3.27 ± 1.39% (Table 4).

## 4. Discussion

In this study, buck semen was collected at the beginning of September, which is approximately 6 weeks prior to the start of the breeding season in Sweden. A protocol was developed that suited the conditions under which we were working. Teasers were brought into estrus, two ejaculates were collected from each buck, a non-egg yolk containing extender was added, the semen was transported, cooled, and the seminal plasma was removed by centrifugation before resuspension to the final concentration for freezing in liquid nitrogen vapor. Post-thaw motility was above 40% (except for two ejaculates), which was considered acceptable for AI involving vaginal deposition of semen. Plasma membrane integrity was lower than sperm motility, in line with other studies [12]. It is not known why the membrane integrity was apparently lower than the motility in both the present study and the previous study by Jiménez-Rabadan et al. [12]. Total sperm numbers were lower than in semen obtained from bucks on Las Palmas [3] but the ejaculates in their study were collected during the breeding season whereas ours were obtained approximately 6 weeks prior to the breeding season. Sperm motility and kinematics were reduced by cryopreservation, in agreement with other studies [2,3]. Sperm morphology was excellent, higher than in the study by Salmani et al. [5].

The timing of semen collection (non-breeding season, approximately 6 weeks prior to the start of the breeding season) in the present study was dictated by the availability of the bucks. There are differing opinions on the effect of out-of-season semen collection on buck sperm quality. Timing of semen collection affected sperm quality in Angora goats but not in Boer goats [13]. However, there may have been an interaction between breed and method of semen collection in the latter study which was not tested in the statistical model. Sperm motility and morphology were better in the breeding season than in the non-breeding season for goats in Greece [14], and for goats in Spain [2]. In contrast, collection of semen in the breeding season versus the non-breeding season was not found to influence a number of parameters of sperm quality for bucks in another study in Spain [4]. 

Collection of semen by artificial vagina was considered to be the most practical method of obtaining good quality semen for this study. The bucks were accustomed to natural mating but quickly adapted to ejaculating into an AV when presented with an estrus doe. Although electroejaculation was used successfully by others [4], it was found to have an adverse effect on sperm motility in a previous study [13]. The latter authors concluded that the AV method was better for collecting semen from Boer goats. Since electroejaculation requires anesthesia and permission from the Board of Agriculture if used in Sweden, it was not considered to be practical for the purposes of this study. 

As mentioned previously, components of buck seminal plasma react with milk-based or egg yolk-based semen extenders, with a negative effect on sperm viability. Therefore, it was previously considered necessary to remove the seminal plasma when such extenders were used for cryopreserving buck semen. However, in an elegant study by Jiminez-Rabadán et al. [4], the effects of removal of seminal plasma on post-thaw sperm quality was examined, in addition to semen collection method, season, and extender. They concluded that although better post-thaw sperm quality could be obtained by collecting ejaculates during the breeding season, and using Andromed or Biladyl rather than skimmed milk extender, centrifugation to remove the seminal plasma did not improve post-thaw sperm quality. Salmani et al. [5] agreed that soy lecithin could substitute for egg yolk in extenders for freezing goat semen. However, removal of seminal plasma was performed in the present study using a similar protocol to [1] and [7]; none of these studies used an extender containing egg yolk or skimmed milk. 

The length of equilibration used here (4 h) was longer than in most other studies, except for that of Jiminez-Rabadán et al. [4]. This was dictated by the travelling time between the farm and the laboratory but also because we chose to collect a second ejaculate from each buck while at the farm, which took approximately one hour after the collection of the first ejaculates. However, there is no indication that the longer equilibration time had a negative effect on post-thaw sperm quality; mean sperm kinematics of the thawed sperm samples were higher than those reported by Nadri et al. [8], who used an equilibration time of 2.5 h. However, there were other differences in the two protocols which might have affected post-thaw sperm quality. A longer equilibration time may have a beneficial effect in allowing stabilization of sperm membranes. It should be noted that some protocols for cryopreserving bull semen include overnight equilibration, or even up to 72 h equilibration, without a detrimental effect on post-thaw sperm quality [15]. There are no reports, to our knowledge, of such a lengthy equilibration being used for buck semen.

The variation in sperm concentration in samples used for freezing in different reports may depend on their intended use. In this study, the intended purpose was for gamete banking, with eventual use in vaginal insemination using the “shot in the dark” deposition technique. In previous studies in Scandinavia, a sperm dose of 200 × 10^6^ per straw was used for vaginal insemination [1]. If the semen is intended for deposition into the uterine horns via a laparoscope or IVF, 50 × 10^6^ spermatozoa per straw (corresponding to a final concentration of 200 × 10^6^/mL) might be more appropriate than the 200 × 10^6^ spermatozoa per straw used here [8].

In a pilot study with two ejaculates collected prior to the study reported here, the semen was kept at room temperature during transport to the laboratory and then cooled for 2 h after centrifugation at room temperature. One ejaculate was prepared at a sperm concentration of 1000 × 10^6^/mL and the other at 800 × 10^6^/mL; the batch with the lower sperm concentration had better post-thaw motility (51% compared to 6%) despite having similar pre-freeze motility. Therefore, a final sperm concentration of 800 × 10^6^/mL was chosen for the present study, to provide 200 × 10^6^ spermatozoa per straw. This concentration was lower than in the previous study involving Norwegian dairy bucks [1] but similar to the concentration reported in a different Norwegian study using the same extender [7]. However, semen was frozen at a controlled rate in the latter two studies whereas vapor freezing over the surface of liquid nitrogen was used in our study.

Vapor freezing was employed for the semen samples in this study, whereas controlled rate freezing was used by Nordstoga et al. [1] and Reiten et al. [7]. The advantage of slow freezing in a programmable machine is that there is a controlled reduction in temperature over the critical range when cell dehydration and ice crystal formation are occurring, allowing equilibration between extracellular and intracellular environments [16]. Thus, theoretically, damage to sperm membranes and mitochondria should be minimized. With vapor freezing, the rate of cooling is determined by the volume of semen, the dimensions of the straw and the height of the rack above the surface of liquid nitrogen [17]. Pellet freezing on dry ice is another option that has been used for goat semen but is less suitable for sperm banking because the pellets take up more space than straws during subsequent storage in liquid nitrogen and are difficult to label for inventory purposes. 

One of the disadvantages of the previous studies comparing various factors likely to affect cryo-survival, such as egg yolk- and non-egg yolk-containing extenders, or semen collection method, or timing of season collection, is that the fertilizing capacity of the thawed sperm samples was not determined, except in the studies by Dorado et al. [2], Chelucci et al. [6] and Nadri et al. [8]. Interestingly, Dorado et al. [2] found that the effect of these various factors was different for semen samples that achieved a higher pregnancy rate than those that were less successful. Chelucci et al. [6] demonstrated that the ability of buck spermatozoa to fertilize sheep oocytes was higher when frozen in 1% lecithin than in egg yolk-containing medium, despite having better motility and membrane integrity in the egg yolk medium. Nadri et al. [8] incorporated an IVF study in their comparison of nano-lecithin, lecithin and egg yolk-based media for buck spermatozoa. They reported that, although post-thaw sperm quality was higher for nano-lecithin than for the other media, there were no significant differences in cleavage or blastocyst development between the extenders. Therefore, attempting to optimize a sperm freezing protocol on analysis of sperm quality without any corresponding data on fertilizing ability may be difficult. Fertilizing ability is the ultimate test of any sperm preservation protocol.

The bucks in our study were of known fertility in natural mating and post-thaw sperm motility was considered to be acceptable; therefore, we have no reason to suppose that the spermatozoa would not be fertile when used in AI [18]. However, one of the problems with semen banking is that the fertilizing capability of the sperm samples is not known until the samples are inseminated [19]. It would be very useful to have an assay that was predictive of fertilization in vivo that could be performed at the same time as a routine post-thaw check. Otherwise, if further straws are thawed to make additional checks of sperm quality, there are no straws left for banking. 

Studies on improving the cryo-survival of buck spermatozoa investigated the addition of substances, such as antioxidants, to the cryomedium. Thus, aloe vera [20], coconut oil [21], crocin [22], glutathione [23], melatonin [24], omega 3, 6 and 9 [25], phenolic compounds [26], and rainbow trout seminal plasma [27] were all considered to improve post-thaw sperm quality. However, it remains to be seen whether fertility is also improved. Colloid centrifugation of buck spermatozoa [12,26] was also reported to have a beneficial effect on post-thaw sperm quality. This technique was previously used for fresh stallion and boar semen and shown to improve pregnancy rates in inseminated mares [28] and sows [29], respectively.

## 5. Conclusions

A practical method for freezing goat semen at a distance from the farm was developed. The semen was extended in warm semen extender containing soy lecithin and was placed immediately in an insulated box with a cold pack for up to 4 h, during transport to the laboratory. Following centrifugation at 4 °C and resuspension in Andromed, straws were filled and subsequently frozen in racks 4 cm above the surface of liquid nitrogen. This simple protocol resulted in an acceptable post-thaw quality for all seven bucks.

## Figures and Tables

**Table 1:** Comparison of various protocols for freezing goat semen.

Reference	Season; Breed	Collection Method	Extender	Removal of Seminal Plasma	Length of Equilibration *	Sperm Concentration	Freezing Method	Post-Thaw Motility
[1]	Non-BS; Norwegian Dairy goat	AV	Andromed	Yes	2.5 h at 5 °C	1000 × 10^6^ (0.25 mL straws)	Controlled rate	Not given
[2]	Non-BS vs. BS; Florida	AV	Egg yolk extender vs. skimmed milk extender	Yes	5 h at 5 °C	250 × 10^6^ rapid progressively motile spz. (0.5 mL straws)	Vapor	48.89–60.72%
[3]	BS; Majorera	AV	Egg yolk	Yes	5 h at 5 °C	400 × 10^6^ (0.5 mL straws)	Vapor	32.6–45.3%
[4]	Non-BS vs. BS; Blanca Celtibérica	EE vs AV	Andromed vs. egg yolk vs. skimmed milk	Removal vs. non-removal	4 h at 5 °C	140–200 × 10^6^ (0.25 mL straws)	Vapor	24–5–5.3% depending on extender and season
[5]	Non-BS; Mahabadi	AV	Tris, citric acid, fructose, glycerol, lecithin vs. egg yolk	No	2.5 h at 5 °C	400 × 10^6^ (0.25 mL straws)	Vapor	31.6–59.8% depending on treatment
[6]	Non-BS; Sarda	AV	Tris, citric acid, glucose, lecithin	No	2 h at 4 °C	400 × 10^6^ Pellet	Dry ice	10–60% depending on treatment
[7]	Not specified: Norwegian Dairy goat	AV	Andromed	Yes	2 h at 5 °C	800 × 10^6^ (0.25 mL straws)	Controlled rate	30.6–36.0%
[8]	BS; Mahabadi	AV	Tris, fructose, citric acid, lecithin vs egg yolk	No	2.5 h at 4 °C	50 × 10^6^ (0.25 mL straws)	Vapor	50–70% depending on treatment

Notes: BS = Breading season; AV = artificial vagina, EE = electroejaculation; spz = spermatozoa. * length of equilibration includes cooling from room temperature to 5 °C and then holding at this temperature. Controlled rate = slow freezing at a controlled rate; vapor = rapid freezing in liquid nitrogen vapor above the surface of liquid nitrogen; pellet = rapid freezing by dropping a set volume of extended semen on to dry ice.

**Table 2:** Characteristics (LSMeans ± sem and Mean ± SD) of buck semen: total number of spermatozoa, initial motility and post-thaw motility (n = 7 bucks).

Buck Number	Total No. Spermatozoa		Initial Motility (%)		Post-Thaw Motility (%)		Difference between Initial and Post-Thaw Motility; %	
	Ejac 1	Ejac 2	Ejac 1	Ejac 2	Ejac 1	Ejac 2	Ejac 1	Ejac 2
1	5540	1780	95	93	84	58	−11	−35
2	6970	4890	93	94	47	73	−46	−21
3	3500	4100	93	80	57	55	−36	−25
4	3610	4240	94	91	0	75	−94	−16
5	3640	2620	93	95	39	59	−54	−36
6	4270	6210	91	92	46	72	−45	−20
7	3610	5000	93	90	66	44	−27	−46
Mean ± SD	4449 ± 1325	4120 ± 1499	93 ± 1	91 ± 5	48 ± 26	62 ± 11		
LSMeans ± sem			93 ± 0.8	94 ± 0.8	48 ± 7.6	62 ± 7.6		
Overall mean ± SD (Batch 1 + Batch 2)	4284 ± 1369		92 ± 4		55 ± 21			

Note: no significant differences detected between ejaculate 1 and 2.

**Table 3:** Sperm kinematics (LSMeans ± sem) for fresh and frozen buck semen (*n* = 14).

	TM (%)	PM (%)	VAP µm/s	VCL µm/s	VSL µm/s	STR	LIN	WOB	ALH µm	BCF Hz
Fresh semen	92 ± 3	85 ± 3	82 ± 1	174 ± 3	57 ± 2	0.69 ± 0.01	0.32 ± 0.01	0.46 ± 0.005	6.0 ± 0.2	24.6 ± 0.7
Frozen semen	59 ± 3	48 ± 3	77 ± 1	145 ± 3	61 ± 2	0.79 ± 0.01	0.42 ± 0.01	0.53 ± 0.005	5.1 ± 0.2	28.3 ± 0.7
*p*	<0.0001	<0.0001	0.02	<0.0001	0.039	<0.0001	<0.0001	0.0001	0.0003	<0.0001

Note: TM = Total motility, PM = progressive motility; VAP = velocity of the average path; VCL = curvilinear velocity; VSL = straight line velocity; STR = straightness; LIN = Linearity; WOB = wobble; ALH = amplitude of lateral head displacement; BCF = beat cross frequency. *p* values shown are the difference between fresh and frozen semen. In addition, there was an interaction between ejaculate and treatment for VCL (*p* = 0.024) and VAP (*p* < 0.029).

**Table 4:** Membrane integrity and DNA fragmentation (LSMeans ± sem) in frozen buck spermatozoa.

Buck Number	Membrane Intact (%)		%DFI	
	Batch 1	Batch 2	Batch 1	Batch 2
1	39	20	2.17	6.04
2	36	48	1.88	3.33
3	25	22	2.72	3.18
4	0	51	2.11	2.86
5	15	28	2.97	3.47
6	20	42	2.4	4.1
7	47	26	2.16	6.36
Mean ± SD4	26 ± 16	34 ± 13	2.34 ± 0.38 *	4.19 ± 1.42 *
LSMeans ± sem	26 ± 5.5	34 ± 5.5	2.34 ± 0.2 *	3.4 ± 0.2 *
Overall mean ± SD for batch 1 and 2	29.9 ± 14.7		3.27 ± 1.39	

Significant difference between ejaculate 1 and ejaculate 2 for both mean ± SD and LSMean ± sem, * ≤ 0.01 for both.

## Data Availability

All data are provided in the article.
